# 3,4-Dihy­droxy­phenethyl acetate

**DOI:** 10.1107/S1600536811026730

**Published:** 2011-07-09

**Authors:** Fei Shen, Jing Zhu, Lu-lu Wang, Kai Wang, Wen-ge Yang

**Affiliations:** a Jiangsu Engineering Technology Research Center of, Polypeptide Pharmaceutical, Nanjing 210009, People’s Republic of China; b State Key Laboratory of Materials-Oriented Chemical Engineering, School of Pharmaceutical Sciences, Nanjing University of Technology, Xinmofan Road No. 5 Nanjing, Nanjing 210009, People’s Republic of China

## Abstract

In the title compound, C_10_H_12_O_4_, the dihedral angle between the acetate group and the aromatic ring is 20.47 (10)°. In the crystal, mol­ecules are linked by O—H⋯O hydrogen bonds, forming [001] chains. Weak C—H⋯O inter­actions consolidate the packing.

## Related literature

For the synthesis, see: Bovicelli *et al.* (2007 ▶).
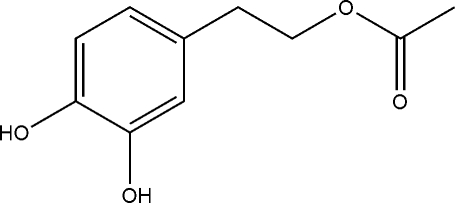


## Experimental

### 

#### Crystal data

C_10_H_12_O_4_
*M* *_r_* = 196.20
Monoclinic, 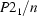

*a* = 11.088 (2) Å
*b* = 7.7100 (15) Å
*c* = 12.687 (3) Å
β = 114.50 (3)°
*V* = 986.9 (3) Å^3^
*Z* = 4
Mo *K*α radiation
μ = 0.10 mm^−1^
*T* = 293 K
0.30 × 0.20 × 0.10 mm

#### Data collection

Enraf–Nonius CAD-4 diffractometer
Absorption correction: ψ scan (North *et al.*, 1968 ▶) *T*_min_ = 0.970, *T*_max_ = 0.990
3672 measured reflections
1819 independent reflections
1439 reflections with *I* > 2σ(*I*)
*R*_int_ = 0.025
3 standard reflections every 200 reflections  intensity decay: 1%

#### Refinement

*R*[*F* ^2^ > 2σ(*F* ^2^)] = 0.046
*wR*(*F* ^2^) = 0.146
*S* = 1.01
1819 reflections
129 parameters
H-atom parameters constrained
Δρ_max_ = 0.28 e Å^−3^
Δρ_min_ = −0.21 e Å^−3^

### 

Data collection: *CAD-4 EXPRESS* (Enraf–Nonius, 1994 ▶); cell refinement: *CAD-4 EXPRESS*; data reduction: *XCAD4* (Harms & Wocadlo, 1995 ▶); program(s) used to solve structure: *SHELXS97* (Sheldrick, 2008 ▶); program(s) used to refine structure: *SHELXL97* (Sheldrick, 2008 ▶); molecular graphics: *SHELXTL* (Sheldrick, 2008 ▶); software used to prepare material for publication: *PLATON* (Spek, 2009 ▶).

## Supplementary Material

Crystal structure: contains datablock(s) global, I. DOI: 10.1107/S1600536811026730/hb5912sup1.cif

Structure factors: contains datablock(s) I. DOI: 10.1107/S1600536811026730/hb5912Isup2.hkl

Supplementary material file. DOI: 10.1107/S1600536811026730/hb5912Isup3.cml

Additional supplementary materials:  crystallographic information; 3D view; checkCIF report

## Figures and Tables

**Table 1:** Hydrogen-bond geometry (Å, °)

*D*—H⋯*A*	*D*—H	H⋯*A*	*D*⋯*A*	*D*—H⋯*A*
O1—H1*A*⋯O2^i^	0.82	2.11	2.827 (2)	145
O2—H2*A*⋯O4^ii^	0.82	1.89	2.7138 (19)	179
C10—H10*A*⋯O1^iii^	0.96	2.36	3.316 (3)	177

Symmetry codes: (i) 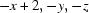; (ii) 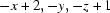; (iii) 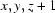.

**Fig. 1.**
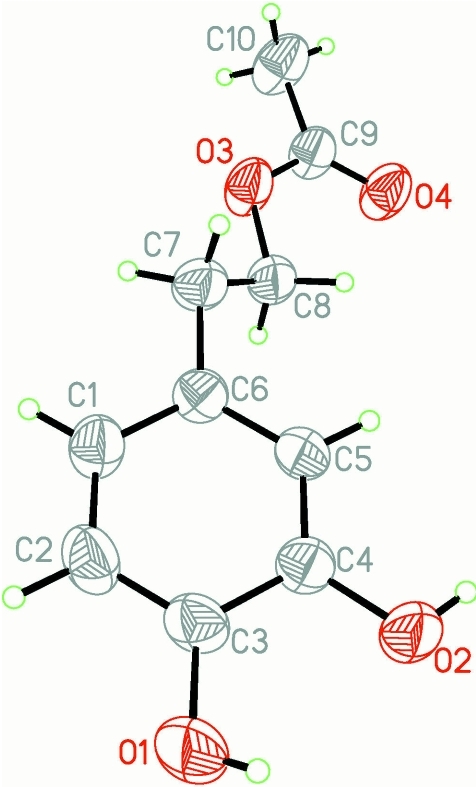
The molecular structure of (I) with displacement ellipsoids drawn at 30% probability levels.

**Fig. 2.**
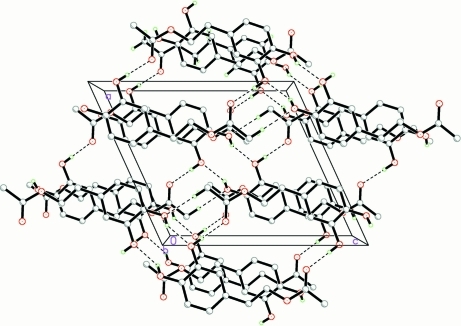
A practical packing diagram of the title compound. Hydron bonds are shown as dashed lines.

## supplementary crystallographic information

### Experimental

The title compound was prepared by the literature method (Bovicelli *et al.* 2007). Colourless blocks of (I) were obtained by slow evaporation of an ethanol solution.

### Refinement

H atoms were positioned geometrically with C—H = 0.93, 0.98 and 0.97 Å for aromatic, methine and methylene H atoms, respectively, and constrained to ride on their parent atoms, with *U*_iso_(H) = 1.2 (or 1.5 for methyl groups) times *U*_eq_(C).

### Crystal data

**Table:**

C_10_H_12_O_4_	*F*(000) = 416
*M**_r_* = 196.20	*D*_x_ = 1.320 Mg m^−^^3^
Monoclinic, *P*2_1_/*n*	Mo *K*α radiation, λ = 0.71073 Å
*a* = 11.088 (2) Å	Cell parameters from 25 reflections
*b* = 7.7100 (15) Å	θ = 9–13°
*c* = 12.687 (3) Å	µ = 0.10 mm^−^^1^
β = 114.50 (3)°	*T* = 293 K
*V* = 986.9 (3) Å^3^	Block, colorless
*Z* = 4	0.30 × 0.20 × 0.10 mm

### Data collection

**Table:**

Enraf–Nonius CAD-4 diffractometer	1439 reflections with *I* > 2σ(*I*)
Radiation source: fine-focus sealed tube	*R*_int_ = 0.025
graphite	θ_max_ = 25.4°, θ_min_ = 2.1°
ω/2θ scans	*h* = 0→13
Absorption correction: ψ scan (North *et al.*, 1968)	*k* = −9→9
*T*_min_ = 0.970, *T*_max_ = 0.990	*l* = −15→13
3672 measured reflections	3 standard reflections every 200 reflections
1819 independent reflections	intensity decay: 1%

### Refinement

**Table:**

Refinement on *F*^2^	Secondary atom site location: difference Fourier map
Least-squares matrix: full	Hydrogen site location: inferred from neighbouring sites
*R*[*F*^2^ > 2σ(*F*^2^)] = 0.046	H-atom parameters constrained
*wR*(*F*^2^) = 0.146	*w* = 1/[σ^2^(*F*_o_^2^) + (0.1*P*)^2^ + 0.110*P*] where *P* = (*F*_o_^2^ + 2*F*_c_^2^)/3
*S* = 1.01	(Δ/σ)_max_ < 0.001
1819 reflections	Δρ_max_ = 0.28 e Å^−^^3^
129 parameters	Δρ_min_ = −0.21 e Å^−^^3^
0 restraints	Extinction correction: *SHELXS97* (Sheldrick, 2008), Fc^*^=kFc[1+0.001xFc^2^λ^3^/sin(2θ)]^-1/4^
Primary atom site location: structure-invariant direct methods	Extinction coefficient: 0.113 (13)

### Special details

**Table:**

Geometry. All e.s.d.'s (except the e.s.d. in the dihedral angle between two l.s. planes) are estimated using the full covariance matrix. The cell e.s.d.'s are taken into account individually in the estimation of e.s.d.'s in distances, angles and torsion angles; correlations between e.s.d.'s in cell parameters are only used when they are defined by crystal symmetry. An approximate (isotropic) treatment of cell e.s.d.'s is used for estimating e.s.d.'s involving l.s. planes.
Refinement. Refinement of *F*^2^ against ALL reflections. The weighted *R*-factor *wR* and goodness of fit *S* are based on *F*^2^, conventional *R*-factors *R* are based on *F*, with *F* set to zero for negative *F*^2^. The threshold expression of *F*^2^ > σ(*F*^2^) is used only for calculating *R*-factors(gt) *etc*. and is not relevant to the choice of reflections for refinement. *R*-factors based on *F*^2^ are statistically about twice as large as those based on *F*, and *R*- factors based on ALL data will be even larger.

### Fractional atomic coordinates and isotropic or equivalent isotropic displacement parameters (Å^2^)

**Table:**

	*x*	*y*	*z*	*U*_iso_*/*U*_eq_	
O1	0.82657 (16)	0.1555 (2)	−0.06613 (11)	0.0744 (5)	
H1A	0.8814	0.0814	−0.0619	0.112*	
C1	0.61846 (18)	0.1594 (2)	0.09171 (14)	0.0500 (5)	
H1B	0.5326	0.1915	0.0787	0.060*	
O2	0.99568 (12)	0.0172 (2)	0.13823 (10)	0.0600 (4)	
H2A	1.0401	−0.0162	0.2045	0.090*	
C2	0.6604 (2)	0.1768 (3)	0.00363 (14)	0.0551 (5)	
H2B	0.6023	0.2195	−0.0681	0.066*	
O3	0.67398 (12)	0.1071 (2)	0.48254 (10)	0.0591 (4)	
C3	0.78691 (19)	0.1315 (2)	0.02105 (14)	0.0493 (5)	
O4	0.85894 (13)	0.0936 (2)	0.64219 (11)	0.0667 (5)	
C4	0.87258 (17)	0.0653 (2)	0.12809 (14)	0.0444 (4)	
C5	0.83054 (17)	0.0485 (2)	0.21595 (13)	0.0435 (4)	
H5A	0.8886	0.0053	0.2875	0.052*	
C6	0.70253 (17)	0.0951 (2)	0.19887 (14)	0.0425 (4)	
C7	0.65213 (17)	0.0689 (3)	0.29153 (14)	0.0498 (5)	
H7A	0.5705	0.1339	0.2703	0.060*	
H7B	0.6312	−0.0529	0.2934	0.060*	
C8	0.74690 (17)	0.1231 (2)	0.41116 (15)	0.0490 (5)	
H8A	0.8242	0.0483	0.4398	0.059*	
H8B	0.7757	0.2418	0.4109	0.059*	
C9	0.73987 (17)	0.0917 (2)	0.59542 (15)	0.0497 (5)	
C10	0.6508 (2)	0.0698 (4)	0.65552 (17)	0.0706 (7)	
H10A	0.6997	0.0906	0.7369	0.106*	
H10B	0.6163	−0.0463	0.6439	0.106*	
H10C	0.5788	0.1508	0.6246	0.106*	

### Atomic displacement parameters (Å^2^)

**Table:**

	*U*^11^	*U*^22^	*U*^33^	*U*^12^	*U*^13^	*U*^23^
O1	0.0785 (10)	0.1125 (13)	0.0404 (7)	0.0258 (9)	0.0327 (7)	0.0154 (7)
C1	0.0448 (9)	0.0593 (11)	0.0435 (9)	0.0051 (8)	0.0162 (8)	−0.0028 (7)
O2	0.0493 (7)	0.0940 (11)	0.0420 (7)	0.0130 (7)	0.0241 (6)	0.0090 (6)
C2	0.0565 (11)	0.0675 (12)	0.0346 (9)	0.0115 (9)	0.0122 (8)	0.0035 (8)
O3	0.0406 (7)	0.1006 (11)	0.0383 (7)	0.0101 (6)	0.0185 (5)	0.0056 (6)
C3	0.0586 (11)	0.0581 (11)	0.0337 (8)	0.0033 (8)	0.0217 (8)	−0.0002 (7)
O4	0.0414 (8)	0.1125 (12)	0.0430 (7)	0.0016 (7)	0.0144 (6)	0.0054 (7)
C4	0.0455 (9)	0.0515 (10)	0.0373 (8)	0.0000 (7)	0.0184 (7)	−0.0019 (7)
C5	0.0462 (9)	0.0501 (9)	0.0344 (8)	0.0020 (7)	0.0169 (7)	0.0041 (7)
C6	0.0436 (9)	0.0458 (9)	0.0386 (9)	−0.0013 (7)	0.0176 (7)	−0.0026 (7)
C7	0.0469 (10)	0.0627 (11)	0.0443 (10)	0.0003 (8)	0.0235 (8)	0.0026 (8)
C8	0.0439 (9)	0.0641 (11)	0.0438 (9)	0.0065 (8)	0.0231 (8)	0.0074 (8)
C9	0.0414 (10)	0.0702 (12)	0.0380 (9)	0.0037 (8)	0.0170 (7)	−0.0026 (8)
C10	0.0529 (12)	0.1190 (19)	0.0459 (11)	−0.0016 (12)	0.0264 (9)	−0.0075 (11)

### Geometric parameters (Å, °)

**Table:**

O1—C3	1.362 (2)	C4—C5	1.381 (2)
O1—H1A	0.8200	C5—C6	1.391 (2)
C1—C6	1.383 (2)	C5—H5A	0.9300
C1—C2	1.384 (2)	C6—C7	1.510 (2)
C1—H1B	0.9300	C7—C8	1.503 (3)
O2—C4	1.368 (2)	C7—H7A	0.9700
O2—H2A	0.8200	C7—H7B	0.9700
C2—C3	1.371 (3)	C8—H8A	0.9700
C2—H2B	0.9300	C8—H8B	0.9700
O3—C9	1.316 (2)	C9—C10	1.487 (2)
O3—C8	1.448 (2)	C10—H10A	0.9600
C3—C4	1.392 (2)	C10—H10B	0.9600
O4—C9	1.202 (2)	C10—H10C	0.9600
			
C3—O1—H1A	109.5	C8—C7—C6	114.80 (14)
C6—C1—C2	120.88 (16)	C8—C7—H7A	108.6
C6—C1—H1B	119.6	C6—C7—H7A	108.6
C2—C1—H1B	119.6	C8—C7—H7B	108.6
C4—O2—H2A	109.5	C6—C7—H7B	108.6
C3—C2—C1	120.62 (16)	H7A—C7—H7B	107.5
C3—C2—H2B	119.7	O3—C8—C7	105.67 (13)
C1—C2—H2B	119.7	O3—C8—H8A	110.6
C9—O3—C8	119.11 (13)	C7—C8—H8A	110.6
O1—C3—C2	119.20 (16)	O3—C8—H8B	110.6
O1—C3—C4	121.50 (17)	C7—C8—H8B	110.6
C2—C3—C4	119.29 (16)	H8A—C8—H8B	108.7
O2—C4—C5	123.78 (16)	O4—C9—O3	122.44 (17)
O2—C4—C3	116.22 (15)	O4—C9—C10	125.14 (16)
C5—C4—C3	119.99 (16)	O3—C9—C10	112.42 (15)
C4—C5—C6	120.92 (16)	C9—C10—H10A	109.5
C4—C5—H5A	119.5	C9—C10—H10B	109.5
C6—C5—H5A	119.5	H10A—C10—H10B	109.5
C1—C6—C5	118.30 (15)	C9—C10—H10C	109.5
C1—C6—C7	119.81 (15)	H10A—C10—H10C	109.5
C5—C6—C7	121.82 (15)	H10B—C10—H10C	109.5
			
C6—C1—C2—C3	−0.5 (3)	C2—C1—C6—C7	−176.88 (17)
C1—C2—C3—O1	−177.86 (17)	C4—C5—C6—C1	−0.2 (3)
C1—C2—C3—C4	0.9 (3)	C4—C5—C6—C7	176.70 (15)
O1—C3—C4—O2	−3.4 (3)	C1—C6—C7—C8	−138.32 (18)
C2—C3—C4—O2	177.79 (17)	C5—C6—C7—C8	44.8 (2)
O1—C3—C4—C5	177.72 (17)	C9—O3—C8—C7	158.48 (17)
C2—C3—C4—C5	−1.0 (3)	C6—C7—C8—O3	173.27 (15)
O2—C4—C5—C6	−178.03 (16)	C8—O3—C9—O4	1.5 (3)
C3—C4—C5—C6	0.7 (3)	C8—O3—C9—C10	−177.75 (18)
C2—C1—C6—C5	0.1 (3)		

### Hydrogen-bond geometry (Å, °)

**Table:**

*D*—H···*A*	*D*—H	H···*A*	*D*···*A*	*D*—H···*A*
O1—H1A···O2^i^	0.82	2.11	2.827 (2)	145
O2—H2A···O4^ii^	0.82	1.89	2.7138 (19)	179
C10—H10A···O1^iii^	0.96	2.36	3.316 (3)	177

Symmetry codes: (i) −*x*+2, −*y*, −*z*; (ii) −*x*+2, −*y*, −*z*+1; (iii) *x*, *y*, *z*+1.
